# The Role of Phospholipid Alterations in Mitochondrial and Brain Dysfunction after Cardiac Arrest

**DOI:** 10.3390/ijms25094645

**Published:** 2024-04-24

**Authors:** Rishabh C. Choudhary, Cyrus E. Kuschner, Jacob Kazmi, Liam Mcdevitt, Blanca B. Espin, Mohammed Essaihi, Mitsuaki Nishikimi, Lance B. Becker, Junhwan Kim

**Affiliations:** 1 Laboratory for Critical Care Physiology, Feinstein Institutes for Medical Research, Northwell Health System, Manhasset, NY 11030, USA; rchoudhary1@northwell.edu (R.C.C.); ckuschner1@northwell.edu (C.E.K.); jkazmi@northwell.edu (J.K.); lmcdevitt@northwell.edu (L.M.); bespin@northwell.edu (B.B.E.); messaihi@umass.edu (M.E.); nishikim@hiroshima-u.ac.jp (M.N.); lance.becker@northwell.edu (L.B.B.); 2 Donald and Barbara Zucker School of Medicine at Hofstra/Northwell, Hempstead, NY 11549, USA

**Keywords:** phospholipids, lysophospholipids, cardiac arrest, ischemia, fatty acids

## Abstract

The human brain possesses three predominate phospholipids, phosphatidylcholine (PC), phosphatidylethanolamine (PE) and phosphatidylserine (PS), which account for approximately 35–40%, 35–40%, and 20% of the brain’s phospholipids, respectively. Mitochondrial membranes are relatively diverse, containing the aforementioned PC, PE, and PS, as well as phosphatidylinositol (PI) and phosphatidic acid (PA); however, cardiolipin (CL) and phosphatidylglycerol (PG) are exclusively present in mitochondrial membranes. These phospholipid interactions play an essential role in mitochondrial fusion and fission dynamics, leading to the maintenance of mitochondrial structural and signaling pathways. The essential nature of these phospholipids is demonstrated through the inability of mitochondria to tolerate alteration in these specific phospholipids, with changes leading to mitochondrial damage resulting in neural degeneration. This review will emphasize how the structure of phospholipids relates to their physiologic function, how their metabolism facilitates signaling, and the role of organ- and mitochondria-specific phospholipid compositions. Finally, we will discuss the effects of global ischemia and reperfusion on organ- and mitochondria-specific phospholipids alongside the novel therapeutics that may protect against injury.

## 1. Introduction to Phospholipids

Phospholipids are crucial components of cell membranes, serving various essential functions in maintaining cellular structure and function. One primary role of phospholipids is to form the lipid bilayer, the basic structural framework of all cell membranes [1]. This bilayer provides a semi-permeable barrier that separates the internal cellular environment from the external surroundings, allowing for compartmentalization and regulation of cellular processes [2,3]. Additionally, phospholipids help maintain membrane fluidity and stability, which are crucial for the proper functioning of membrane-bound proteins, ion channels, and transporters. By modulating the fluidity of cell membranes, phospholipids also influence cell signaling, adhesion, and migration processes. Phospholipids also serve as an energy reservoir, thereby aiding in maintaining energy homeostasis and executing essential metabolic functions [4]. Moreover, phospholipids assume critical functions across various organ systems, notably in the nervous system, where they are indispensable for the formation and functionality of myelin sheaths [5]. These sheaths serve to insulate nerve fibers, facilitating the rapid conduction of nerve impulses.

Beyond their intracellular roles, phospholipids play integral roles in systemic physiological processes within multicellular organisms. Notably, phospholipids are vital constituents of lipoproteins [6], which serve as carriers for lipid transport throughout the bloodstream. Lipoproteins, including low-density lipoprotein and high-density lipoprotein, facilitate the transport of cholesterol and other lipids to and from peripheral tissues, thereby contributing significantly to lipid metabolism and overall lipid homeostasis [7].

Metabolites of phospholipids also play important roles in cell signaling, intracellular communication, and regulation of membrane protein function and various cellular processes. For example, phosphatidylinositol (PI) is a precursor for second messengers involved in signal transduction cascades, such as phosphatidylinositol 4,5-bisphosphate (PIP2). PIP2 is cleaved by phospholipase C to generate inositol trisphosphate and diacylglycerol, which regulate intracellular calcium levels and protein kinase C activation, respectively [8]. Lysophosphatidic acid (LPA) can activate intracellular signaling pathways involved in promoting cell proliferation, survival, and adipocyte differentiation, thereby influencing overall energy metabolism and adipose tissue function [9]. Furthermore, the fatty acids of phospholipids serve as critical precursors for numerous biologically active molecules via enzymatic and non-enzymatic pathways [10,11]. Released fatty acids also serve as substrates for enzymatic reactions, generating bioactive lipid species involved in cell signaling, inflammation, and immune regulation [12]. Thus, the fatty acid components of phospholipids play multifaceted roles in the body’s physiological processes, beyond their structural functions.

Overall, phospholipids are indispensable molecules crucial for maintaining the function, structure, and regulation of cells and tissues throughout the body. The multifaceted functions provided by phospholipids underscore their significance in preserving cellular homeostasis and supporting myriad physiological processes. Notably, phospholipids play a pivotal role in cellular and mitochondrial physiology. Alterations in these functions can implicate various diseases; for instance, oxidized phospholipids are associated with atherosclerosis and non-alcoholic steatohepatitis [13]. Diseases such as cancer, diabetes, cardiovascular, and infectious diseases have been linked to changes in phospholipid composition, distribution, and metabolism [4,5]. Additionally, defects in nuclear genes can lead to phospholipid metabolism disorders, particularly in mitochondrial diseases like Barth syndrome and dilated cardiomyopathy [14,15]. Recent investigations, including our own study, suggest that alterations in phospholipids, particularly within mitochondria, may significantly contribute to brain dysfunction following cardiac arrest [16,17,18,19]. This review aims to explore the potential involvement of mitochondrial phospholipids in neural damage subsequent to cardiac arrest, with the objective of enhancing our comprehension of the pathophysiological mechanisms driving post-cardiac arrest brain injury.

## 2. The Structure of Phospholipids

**Figure 1:**
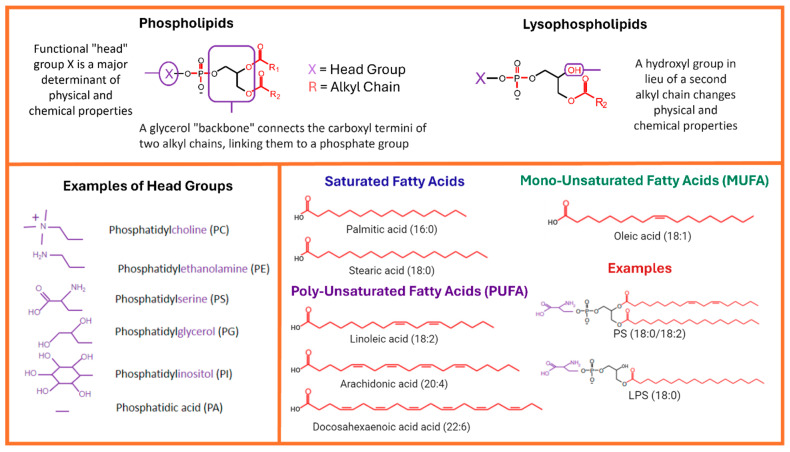
Diagram of phospholipid structure and classification. This diagram illustrates the basic structural features of fatty acids, distinguishing features of phospholipids and lysophospholipids, and provides relevant examples of head groups and major fatty acid chains.

The structure of phospholipids is essential to their function in biological systems. PLs are amphipathic molecules containing both a hydrophilic region and two hydrophobic acyl chains attached to a glycerol molecule serving as a backbone [20]. Lysophospholipids, resulting from the hydrolysis of phospholipids, feature a singular fatty acid chain and serve a pivotal role in maintaining phospholipid homeostasis. The hydrophilic region comprises a phosphate group (-PO4) and a head group that determines the class of phospholipids [20] (Figure 1). The simplest class of phospholipid is phosphatidic acid, which does not carry any head group. If the head group attached to the phosphate moiety is choline, this phospholipid class is called phosphatidylcholine (PC), the most abundant class of phospholipid. Other head groups include ethanolamine, serine, and inositol, and these phospholipids are called phosphatidylethanolamine (PE), phosphatidylserine (PS), and PI, respectively. All head groups are hydrophilic and interact with water molecules.

Two acyl chains derived from fatty acids are attached to the first and the second carbons of the glycerol moiety, which are denoted, respectively, as the sn-1 and sn-2 positions and determine the species of phospholipids within a class. Previous studies have identified common acyl chains of phospholipids [21,22]. For example, stearic acid (18:0), palmitic acid (16:0), and myristic acid (14:0) are major acyl chains of saturated fatty acid, oleic acid (18:1) is the major monounsaturated fatty acid (MUFA), and linoleic acid (18:2), arachidonic acid (20:4), and docosahexaenoic acid (22:6) are major polyunsaturated fatty acids (PUFAs) attached to phospholipids. Other frequently encountered phospholipid fatty acids include (16:1), (22:4), and (20:5). Typically, saturated fatty acids or monounsaturated fatty acids (MUFAs) are present at the sn-1 position and PUFAs at the sn-2 position of phospholipids [23]. Phospholipids containing MUFAs at the sn-2 position are also found. The relative contents of species containing PUFAs and species containing MUFAs at the sn-2 position determine the abundance of PUFAs in a tissue [24].

The structure of phospholipids gives them an amphophilic nature, with hydrophilic head groups and hydrophobic tails, which allows PLs to form lipid bilayers in aqueous environments, such as cell membranes, thus forming a stable barrier that separates the interior of cells from their external surroundings. Phospholipids contribute to the characteristic inner and outer membranes that give them their shape [19]. Moreover, diverse combinations of head groups and fatty acids give rise to a multitude of phospholipid species, each exhibiting unique properties and functions in biological processes and membrane integrity.

## 3. The Reactivity of PUFAs, Their Metabolites, and Their Roles

The degree of fatty acid saturation alters the phospholipid properties. To increase the PUFA concentration, desaturase enzymes can increase unsaturation in fatty acid precursors produced via fatty acid synthase or cell importation [25]. From this point, unique combinations of desaturation, elongation, and beta oxidation can be formed to acquire specific PUFAs [26].

The degree of unsaturation alters phospholipid fluidity, with increased unsaturation increasing membrane fluidity and permeability [27]. Modification of the acyl chains contained in the phospholipids alters membrane functionality, increasing or decreasing the content of PUFAs versus MUFAs, a factor that has been theorized to drastically alter mitochondrial functionality and contribute to disease processes and aging [28]. Furthermore, the degree of unsaturation alters signaling potential, increasing or decreasing the concentrations of various biologically relevant PUFAs, which can be metabolized to biologically potent molecules called eicosanoids and specialized proresolving mediators [29].

Increased membrane unsaturation theoretically increases the number of bisallyic carbons, which have increased reactivity toward ROS and oxidation enzymes, enhancing membrane susceptibility to oxidative modifications [30,31]. ROS-mediated oxidation is especially relevant for mitochondrial phospholipids that border the electron transport chain, the major site of ROS generation [32,33].

Oxidative modifications of PUFAs represent a critical aspect with significant implications for both health and disease. Under conditions of oxidative stress, PUFAs are susceptible to attack by free radicals, initiating a process known as lipid peroxidation [34]. This cascade of events can result in the generation of lipid peroxides, a phenomenon implicated in various pathological processes [35]. Particularly concerning are the by-products of lipid peroxidation, such as malondialdehyde (MDA) and 4-hydroxynonenal (4-HNE), which have been associated with oxidative stress and inflammation, exacerbating tissue damage and contributing to the progression of diseases [36].

Moreover, enzymes including cytochrome P450 oxidases, lipoxygenases, and cyclooxygenases play pivotal roles in mediating the oxidation of PUFAs. These enzymatic pathways lead to the synthesis of bioactive lipid mediators with diverse physiological and pathological functions. For instance, some lipid mediators derived from PUFAs, such as prostaglandins and leukotrienes, are crucial regulators of inflammation and immune responses [37,38]. Their production and subsequent actions are tightly regulated, playing pivotal roles in orchestrating the body’s response to injury and infection, as well as in maintaining tissue homeostasis. Additionally, enzymatic modifications of 22:6 yield lipid mediators, such as protectins, resolvins, and maresins, which play roles in inflammation resolution and neuroprotection [39].

Understanding the interplay between oxidative modifications of PUFAs and the enzymatic processes that mediate their metabolism is paramount for deciphering their roles in health and disease. These processes are intricately involved in the regulation of inflammation, cell signaling, and various other biological functions. Furthermore, dysregulation of these pathways can contribute to the pathogenesis of numerous diseases, ranging from inflammatory disorders to neurodegenerative conditions. Thus, elucidating the mechanisms underlying PUFA oxidation and its consequences is essential for developing targeted therapeutic interventions aimed at mitigating oxidative-stress-related damage and preserving tissue function.

## 4. Lower Phospholipid PUFA Contents in the Brain Compared to Other Tissues

The majority of the phospholipid species found in the body contain PUFAs, and these PUFA-containing phospholipids are distributed across almost every major tissue. However, the large presence of PUFAs has previously been emphasized only in the brain. This has led to the belief that the phospholipid profile of the brain contains more PUFAs, such as 20:4 and 22:6, than other tissues [40,41]. As a result, it has previously been hypothesized that the abundance of these easily oxidized PUFAs is a major component in the susceptibility of the brain to ischemia/reperfusion injury [42]. However, when the phospholipid compositions of brain, heart, liver, and kidney tissues were compared in rats, brain tissue was actually revealed to contain a relatively higher concentration of MUFAs and saturated fatty acids and lower concentration of PUFAs. The PUFA-containing phospholipid species were found to make up only ~60% of the total phospholipid content in the brain, compared to over 90% in the heart and kidney and ~95% in the liver. Furthermore, the specific composition of PC within the brain was observed to be unique when compared to other tissues. Most of the PC species observed in heart, kidney, and liver tissues contained PUFAs, whereas ~65% of total PC content in the brain did not contain any PUFAs. These findings indicate that the brain has a lower relative number of PUFA-containing phospholipids than major organs such as the heart, kidney, and lungs.

In addition, the brain also utilizes a unique profile of PUFAs and MUFAs. Both 22:6 and 20:4 contribute to a significant portion of the total PUFA content in the brain, whereas 20:4 is the primary PUFA present in other tissues. This is consistent with the knowledge that dietary omega-3 fatty acids, such as 22:6, are important for proper brain function [43,44]. Moreover, 18:2 is another dietary fatty acid that plays a crucial role in humans. This omega-6 fatty acid is one of the two essential fatty acids for humans, and it is the required building block for 20:4, prostaglandin, leukotriene, and thromboxane synthesis. Interestingly, despite playing a crucial role in the synthesis of many signaling molecules, 18:2 is noticeably scarce in the brain. However, it is still unknown whether the lack of 18:2 in brain tissue holds any physiological significance.

The major MUFA species present in mammals is 18:1, and this molecule is uniquely abundant in the brain as compared to surrounding tissues [24]. While the importance of other species in brain function, predominately 22:6, has been extensively characterized, the role of 18:1 in maintaining brain function has yet to be elucidated. Although 18:1 is a MUFA, it displays characteristics of both saturated fatty acids and PUFAs. The one double bond in the 18-carbon chain contributes to the curvature of membrane structures [45], similar to PUFAs. However, since 18:1 lacks the bis-allylic position found in all PUFAs, 18:1 is much more stable and resistant to oxidation. Whereas saturated fatty acids and PUFAs are typically bound to the sn-1 and sn-2 positions on glycerol, respectively, 18:1 is capable of binding to both positions. It has been theorized that the ability of 18:1 to bind to both positions may play a significant role in the brain’s response to oxidative stress.

Lastly, the phospholipid profile in the brain is less affected by the lipid content of the diet than other tissues [46,47]. While most tissues reflect dietary changes in the lipid intake of typical fatty acid sources, such as free fatty acids or glycerides, the brain appears to maintain a relatively stable composition, particularly regarding its PUFA concentration. Only the dietary intake of lysophosphatidylcholine (LPC) has been observed to enhance the content of PUFAs in the brain [48]. Neurodegenerative diseases such as Alzheimer’s have been shown to have decreased PUFA content in the brain. While the exact role and significance of the decreased PUFA content in the disease process remain unknown, LPC supplementation may provide a pathway to increase the PUFA content in the brain and counteract neurodegeneration.

## 5. Higher PUFA Contents in Mitochondrial Phospholipids Than Whole Tissue

Phospholipid metabolism in mitochondria is a tightly regulated process that can have significant effects on overall health. Cardiolipin (CL) is a phospholipid unique to the inner mitochondrial membrane (IMM), and proper synthesis of this molecule from PG is crucial for maintaining mitochondrial stability and function. Impaired CL synthesis can lead to conditions such as cardiomyopathy or Barth syndrome [49]. CL only makes up a small percentage of the IMM, with PC (~40%) and PE (~30%) comprising the majority of inner and outer mitochondrial membranes. PI (~10–15%) and PS (~5%) are also present in mitochondria to a lesser extent, although brain mitochondria have been observed to contain higher quantities of PS than other major tissues [21,50]. Mitochondria also contain large amounts of PG, comprising the majority of whole-tissue PG stores. It has been theorized that since PG is the precursor to CL, large quantities are essential for maintaining proper mitochondrial health [51].

**Figure 2:**
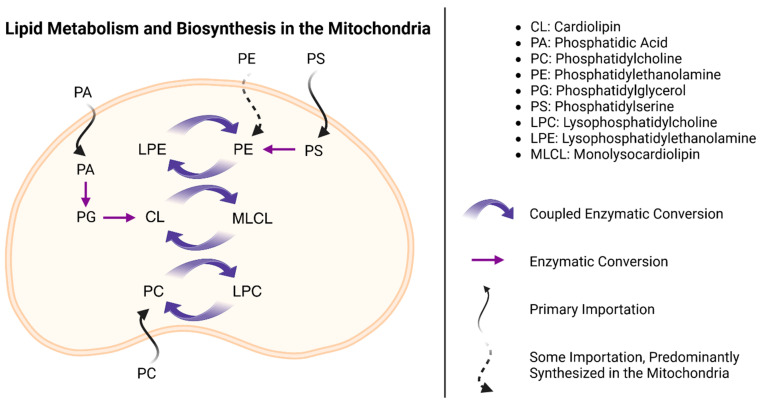
Schematic of phospholipid biosynthesis in the mitochondria. This schematic outlines the importation patterns of precursor phospholipids into the mitochondria, the biosynthetic pathways responsible for converting them into other phospholipids, and the role of cardiolipin in facilitating phospholipid remodeling.

Phospholipid synthesis in mitochondria generally follows one of two possible pathways. Phospholipid species such as PE and PG can be imported or synthesized directly within the mitochondria, whereas PC and PS must first be synthesized in the endoplasmic reticulum before transportation into the mitochondria [52]. CL is also synthesized directly in mitochondria, like PE and PG, but it undergoes a unique remodeling step that can differ slightly depending on the tissue in which it is made (Figure 2).

CL metabolism is unique in the brain when compared to the rest of the body. CL synthesis occurs in mitochondria, where the final structure of CL is determined by a remodeling step. In most tissues, the remodeling step preferentially incorporates 18:2 into CL [53,54]. As a result, CL(18:2)_4_ is found to be the dominant species in most mammalian tissues except for the brain [55]. The brain contains a relatively low concentration of 18:2 and has been observed to synthesize a diverse spectrum of CL species. These observations indicate that the local profile and relative availability of fatty acids may be an important factor in determining CL species. It is also possible that brain mitochondria preferentially limit the usage of 18:2 for the synthesis of CL, instead relying on other available fatty acids, which results in the highly diverse CL spectrum observed in the brain. This possibility is supported by the knowledge that the CL composition in the liver is readily affected by the fat contents in the diet [56,57,58]. The unique profile of CL within the brain suggests that preserving CL(18:2)_4_ is not important for brain mitochondria. This would explain why brain function is not affected in Barth syndrome patients, who have a defective metabolism for the use of 18:2 CL.

The phospholipid composition of mitochondria generally mimics the whole-tissue profile; however, one major difference that has been observed is an increase in PUFA content relative to the rest of the tissue. Mitochondria from brain, heart, kidney, and liver tissues were all observed to contain increased PUFAs relative to the whole-tissue profile [21]. The increased PUFA content is most pronounced in the PC species, one of the largest contributors to the mitochondrial membrane. While PG, PI, and PS have one common dominant species regardless the tissue type, PC is comprised of a variety of species containing both PUFAs and non-PUFAs. This diversity may provide room for PC to change its composition based on the cell type the mitochondria inhabit. For optimal cellular functioning, mitochondrial membranes may require higher concentrations of PUFA-containing phospholipids than non-mitochondrial membranes.

When comparing heart, kidney, liver, and brain tissues, brain mitochondria showed the most significant differences compared to their whole-tissue phospholipid profile. While all of these mitochondria were revealed to have increased levels of PC species containing 22:6 and 20:4, only brain mitochondria were also found to contain PE species that were enriched in PUFAs as well. The PC and PE species in brain mitochondria had ~17% and ~20% greater PUFA contents, respectively, than whole brain tissue [21]. While the PE species in whole brain tissue still had lower overall levels of PUFAs when compared with heart, kidney, and liver PE, the PUFA-containing PE species in brain tissue were uniquely concentrated in the mitochondria. Kidney and liver mitochondria were also observed to contain a significant increase in relative unsaturation in their PC species, while this increase was comparatively minimal in heart mitochondria. Although brain mitochondria had the most significant increase in PUFA content when compared to their whole-tissue profile, the relative PUFA content in brain mitochondria was still found to be the lowest out of all four tissues studied.

It remains unclear why mitochondria require a higher concentration of PUFAs than surrounding tissue membranes. Previously, studies have theorized that mitochondria should preferentially decrease the PUFA content to protect against reactive oxygen species generation [32,59,60]. As PUFAs are prone to oxidative modification and mitochondria represent a major site of ROS generation, it is easy to assume that mitochondria will benefit from decreasing the relative content of PUFAs to limit damage to both the mitochondrial membrane and protein degradation. It must be recognized that factors such as membrane fluidity or the downstream utility of PUFA metabolites are essential components of membrane function that need to be balanced alongside the potential risk of oxidative stress.

## 6. Mitochondrial Phospholipid Modifications and Brain Vulnerability to Cardiac Arrest

The brain, with its intricate network of neurons and delicate cellular architecture, is exceptionally vulnerable to the deleterious effects of ischemia/reperfusion injury, a phenomenon observed in both localized incidents like ischemic strokes and systemic insults such as cardiac arrest and hypotension [61,62]. Indeed, cardiac arrest, which induces whole-body ischemia and affects all organs simultaneously, serves as a pertinent disease model to demonstrate the brain’s vulnerability to ischemia/reperfusion injury compared to other organs. While investigating the precise mechanism underlying the high vulnerability of the brain, previous studies have focused on the detrimental consequences of elevated lysophospholipids and free fatty acids, highlighting their adverse impacts on cerebral function [63]. One prevailing hypothesis suggests that this observed harm arises from the compromise of membrane integrity following a reduction in phospholipid levels, a scenario that may precipitate irreversible brain injury upon the rapid oxidative modification of PUFAs [25,64,65]. This interpretation is based on the belief that the brain is especially enriched in PUFAs [66]. Contrary to conventional belief, the brain does not possess a significantly higher abundance of PUFAs compared to other tissues. This suggests that the proposed mechanism involving oxidative modification of PUFAs may not fully elucidate the brain’s vulnerability to ischemic insult [67].

As previously discussed, PUFAs are indispensable for physiological brain function. Given the brain’s minimal fat storage capacity and its inability to synthesize PUFAs, a consistent supply from the bloodstream is crucial to maintain physiological PUFA levels. Disruption of this essential supply has been shown to result in significant brain dysfunction [68]. Consequently, disturbances in the intake and/or further metabolism of these PUFAs will disproportionately impact the brain, potentially serving as a contributing factor to the heightened vulnerability of the brain to ischemic insult following cardiac arrest.

In our rat model of cardiac arrest, despite marked observed brain damage, there was no significant change in the content or composition of phospholipids in the whole brain tissue [16]. We found an increase in LPC, lysophosphatidylethanolamine (LPE), and lysophosphatidylinositol (LPI). However, the increase in these lysophospholipids was not sufficient to decrease the concentration of phospholipids in whole brain tissue following 30 min CA. Notably, we found that the concentration of CL was decreased by 33% in brain mitochondria [18]. This reduction in brain mitochondria was accompanied by diminished PE and PG contents [69], which are believed to be synthesized in the mitochondria.

Furthermore, a shift was observed in mitochondrial species of PE, PC, and PS from unsaturated to saturated [69], reflecting ratios observed in non-mitochondrial PE, PC, and PS. These data provide two potential mechanisms for mitochondrial phospholipid alterations in the brain, the increased vulnerability of PUFAs to oxidation, and the inability of PUFAs to maintain physiological mitochondrial membrane form and function. Unlike other key organs, the brain does not maintain a robust storage of fatty acids and relies primarily on the replenishment of PUFAs from the systemic circulation [70], which may be compromised after cardiac arrest due to decreased plasma lipid concentrations [71]. In the setting of global ischemia and reperfusion, when widespread multi-organ ischemia leads to diffuse cellular membrane disruption, key organs such as the liver, which normally supply circulatory plasma lipids, may shift towards primarily repairing their own damage instead of synthesizing and distributing essential precursor phospholipids. These features are further compounded by impaired circulation and blood flow to the brain. Taken together, these alterations found only in mitochondrial phospholipids serve as evidence of a potential molecular cause for mitochondrial dysfunction that can produce critical neurological deficits [69].

## 7. Future Directions and Therapeutic Applications

The brain is home to a variety of cellular populations including neurons and glial cells, such as astrocytes, microglia, and oligodendrocytes. Each cell type is composed of different phospholipid makeups, with further variation at the organelle level. Among these organelles, mitochondria have a significant potential to contribute to ischemic-/reperfusion-induced damage due to their contribution to free radical generation, induction of pro-inflammatory signaling, and capacity to induce post-stress cell death.

Phospholipids play an essential role in normal physiological health. From a cellular level, the selection of specific phospholipid compositions facilitates selective cell membrane composition, facilitating critical functions including signaling, membrane trafficking, and metabolism. The organelle-specific phospholipid composition plays a critical role in mitochondrial physiology, with selective upregulation of PUFAs and the maintenance of mitochondria-specific PLs such as CL. Indeed, the maintenance of such a distinct phospholipid composition further demonstrates the unique functions and structural changes that the mitochondrion is required to the execute to maintain its physiological role. Organ-specific mitochondrial phospholipid selection facilitates adequate fluidity, signaling, and response to oxidative exposure. Inborne errors of metabolism such as Barth syndrome demonstrate the importance of maintaining careful regulation of phospholipid synthesis and metabolism. Conversely, a wide variety of disease states stem from or result in phospholipid abnormalities. These range from primary neurological disorders, including Alzheimer’s disease and depression, to metabolic states such as obesity and non-alcoholic fatty liver disease. Alterations in phospholipid metabolism are implicated in a wide variety of inflammatory disease states requiring further research into the therapeutic effects of phospholipid modulation. Furthermore, external stressors such as sepsis, hyperthermia, and ischemia/reperfusion through cardiac arrest cause significant modulation in phospholipid composition and metabolism and are an essential component of the induced pathophysiological state.

Despite the demonstrated therapeutic potential in targeting phospholipid dysregulation or directly supplementing phospholipids for various diseases [72,73,74], their clinical application remains limited. This limitation may arise from challenges in understanding the mechanistic intricacies and controlling phospholipids for experimental design, attributed to the complexity of the metabolic pathways involved [75]. However, recognizing the inherent protective effects of phospholipids against diseases, particularly cardiovascular conditions, underscores the need for nuanced approaches to fully comprehend the role of phospholipids in health and disease management.

## 8. Conclusions

Mitochondria play a vital role in cellular physiology but undergo significant changes in phospholipid structure and metabolism following pathophysiological conditions such as cardiac arrest. These alterations in phospholipid composition affect membrane function and mitochondrial structure. Moreover, coupled with low metabolic stores in the brain, they lead to a diminished supply and ability to replenish essential phospholipid precursor components post-cardiac arrest. Whether these changes primarily cause or result from cellular pathology necessitates further investigation. However, recent research indicates that supplementation with phospholipids can help mitigate these pathological cellular changes and improve outcomes after neural ischemia. These findings underscore the importance of further research not only into the impact of disease states on phospholipids but also into the potential therapeutic effects of modulating organ-specific phospholipid metabolism.
